# Supplementary material for: Within-species phylogenetic relatedness of a common mycorrhizal fungus affects evenness in plant communities through effects on dominant species
Source: PLoS One. 2018 Nov 21;13(11):e0198537. doi: 10.1371/journal.pone.0198537 (PMC6248901; doi:10.1371/journal.pone.0198537)

Table A. Coefficients of linear regressions between relative contribution of each plant species to total aboveground dry mass and plant community evenness.

Table A

Plant species	Coefficient	SE	P-value
A.elatius	24.65	6.41	<0.001
F.pratensis	-105.27	-19.77	<0.001
T.pratense	48.94	5.69	<0.001
L.corniculatus	15.19	2.38	<0.001
K.arvensis	11.25	1.49	<0.001
P.vulgaris	5.54	0.8	<0.001

Note: Roots straining and colonization measurement protocol

The staining protocol was as follow, the roots of all species were rinsed with clean water and transfer in a 2ml tube that was filled with 10% KOH and heated at 90°C for 1h for the roots of *T. pratense, A. elatius, F. pratensis* and *P. vulgaris*, 45min for *K. arvensis* and 30 min for *L. corniculatus*. During the heating time the KOH was change at least three times. At the end of the heating time, the KOH was removed and HCL 1% was added for 3 to 5 minutes. Trypan blue solution was then added either for overnight staining or for a 2h staining at 90°C. The trypan blue was then removed and replaced by lactic acid at 80% for permanent conservation. Roots of each of the 900 plants were randomly spread on a petri dish previously marked with 100 cells of 4x4mm arranged in a grid pattern. The presence or absence of fungal structure was recorded on each cell containing a root and sums up to calculate the colonization level (%).

Fig A. Relationships between ADM responsiveness and AMF colonization rate for each of the six plant species. See main text for statistics

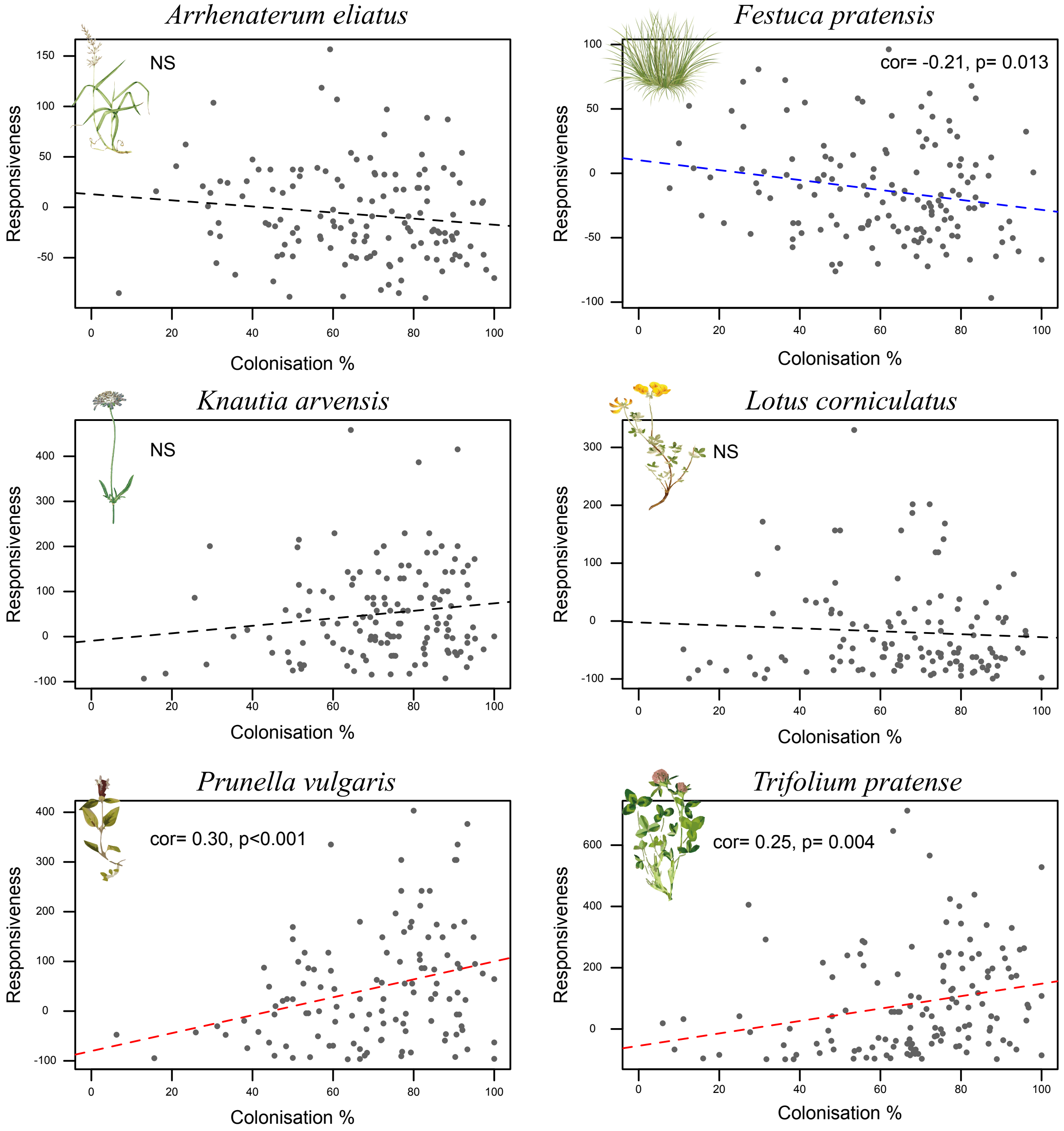

Fig B. Plant community metric for all the treatments and NM for **(1)** ADM and RDM, **(2)** mesocosm productivity (TDM) and **(3)** plant community evenness

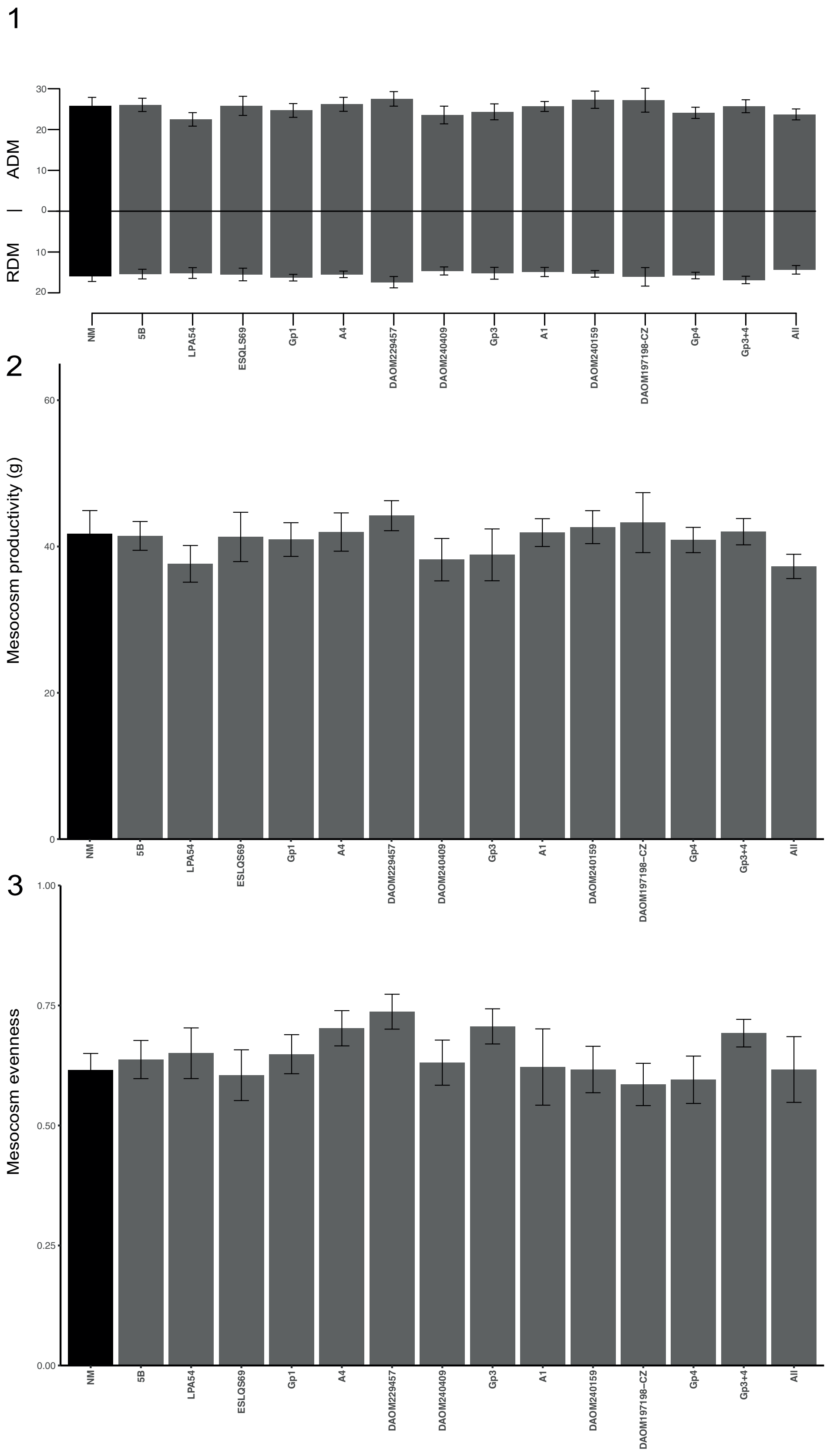

Fig C. Relationship between plant community evenness and mean colonization rate for each mesocosm. See main text for statistics.

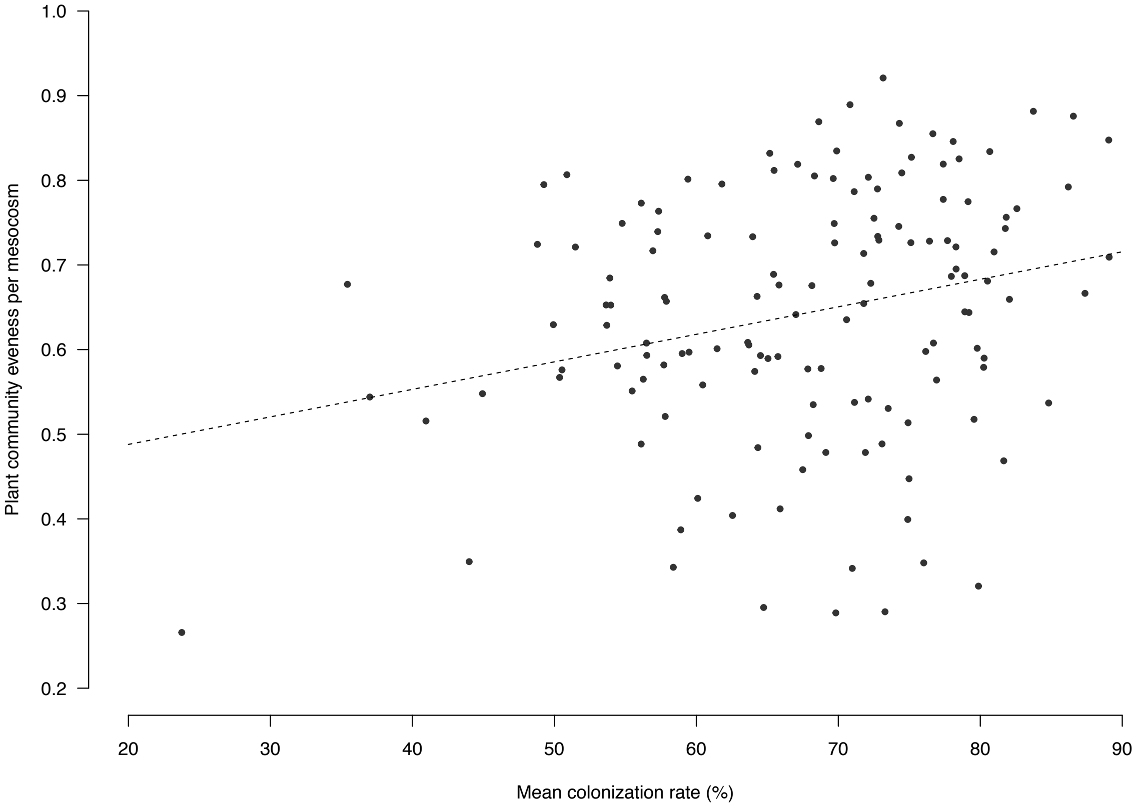

Fig D. Relationship between colonization evenness and mean colonization rate for each mesocosm. See main text for statistics.

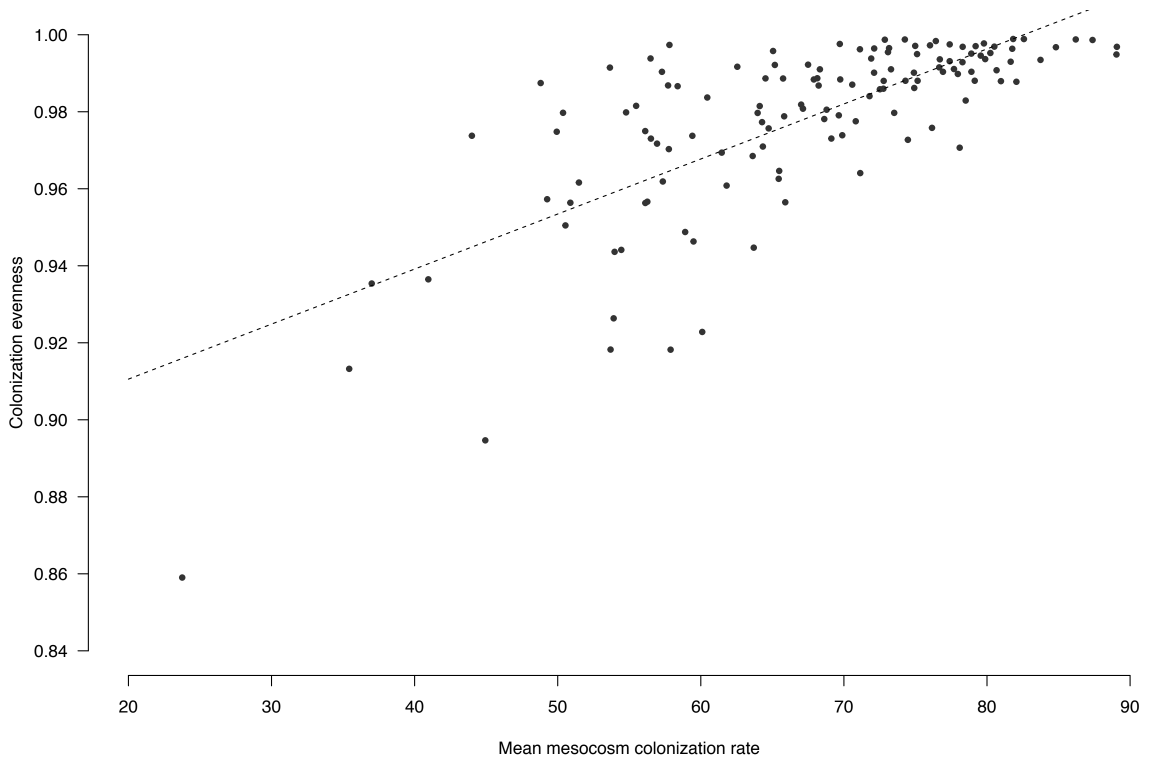

Supplement: S1 File — (PDF) [file pone.0198537.s003.pdf]
